# Patient-reported quality indicators for osteoarthritis: a patient and public generated self-report measure for primary care

**DOI:** 10.1186/s40900-016-0019-x

**Published:** 2016-03-17

**Authors:** Steven Blackburn, Adele Higginbottom, Robert Taylor, Jo Bird, Nina Østerås, Kåre Birger Hagen, John J. Edwards, Kelvin P. Jordan, Clare Jinks, Krysia Dziedzic

**Affiliations:** 1 Arthritis Research UK Primary Care Centre, Research Institute for Primary Care & Health Sciences, Keele University, Keele, UK; 2 Lay Member of the Osteoarthritis Research User Group, Arthritis Research UK Primary Care Centre, Research Institute for Primary Care & Health Sciences, Keele University, Keele, UK; 3 Diakonhjemmet Hospital, Oslo, Norway

**Keywords:** Osteoarthritis, Quality indicator, Patient-reported, Patient and public involvement, Primary care, Impact

## Abstract

**Plain English summary:**

People with osteoarthritis desire high quality care, support and information. However, the quality of care for people with OA in general practice is not routinely collected. Quality Indicators can be used to benefit patients by measuring whether minimum standards of quality care are being met from a patient perspective.

The aim of this study was to describe how a Research User Group (RUG) worked alongside researchers to co-produce a set of self-reported quality indicators for people with osteoarthritis when visiting their general practitioner or practice nurse (primary care). These were required in the MOSAICS study, which developed and evaluated a new model of supported self-management of OA to implement the NICE quality standards for OA.

This article describes the public involvement in the MOSAICS study. This was 1) the co-development by RUG members and researchers of an Osteoarthritis Quality Indicators United Kingdom (OA QI (UK)) questionnaire for use in primary care, and 2) the comparison of the OA QI (UK) with a similar questionnaire developed in Norway.

This study shows how important and effective a research user group can be in working with researchers in developing quality care indicators for osteoarthritis for use in a research study and, potentially, routine use in primary care. The questionnaire is intended to benefit patients by enabling the assessment of the quality of primary care for osteoarthritis from a patient’s perspective. The OA QI (UK) has been used to examine differences in the quality of osteoarthritis care in four European countries.

**Abstract:**

**Background**

People with osteoarthritis (OA) desire high quality care, support and information about OA. However, the quality of care for people with OA in general practice is not routinely collected. Quality Indicators (QI) can be used to benefit patients by measuring whether minimum standards of quality care (e.g. NICE quality standards) are being met from a patient perspective. A Research User Group (RUG) worked with researchers to co-produce a set of self-report, patient-generated QIs for OA. The QIs were intended for use in the MOSAICS study, which developed and evaluated a new model of supported self-management of OA to implement the NICE guidelines. We report on 1) the co-development of the OA QI (UK) questionnaire for primary care; and 2) the comparison of the content of the OA QI (UK) questionnaire with a parallel questionnaire developed in Norway for the Musculoskeletal Pain in Ullensaker (MUST) study.

**Methods**

Researchers were invited to OA RUG meetings. Firstly, RUG members were asked to consider factors important to patients consulting their general practitioner (GP) for OA and then each person rated their five most important. RUG members then discussed these in relation to a systematic review of OA QIs in order to form a list of OA QIs from a patient perspective. RUG members suggested wording and response options for a draft OA QI (UK) questionnaire to assess the QIs. Finally RUG members commented on draft and final versions of the questionnaire and how it compared with a translated Norwegian OA-QI questionnaire.

**Results**

RUG members (5 males, 5 females; aged 52–80 years) attended up to four meetings. RUG members ranked 20 factors considered most important to patients consulting their GP for joint pain. Following discussion, a list of eleven patient-reported QIs for OA consultations were formed. RUG members then suggested the wording and response options of 16 draft items – four QIs were split into two or more questionnaire items to avoid multiple dimensions of care quality within a single item. On comparison of this to the Norwegian OA-QI questionnaire, RUG members commented that both questionnaires contained seven similar QIs. The RUG members and researchers agreed to adopt the Norwegian OA-QI wording for four of these items. RUG members also recommended adopting an additional seven items from the Norwegian OA-QI with some minor word changes to improve their suitability for patients in the UK. One other item from the draft OA QI (UK) questionnaire was retained and eight items were excluded, resulting in a 15-item final version.

**Conclusions**

This study describes the development of patient-reported quality indicators for OA primary care derived by members of a RUG group, working in partnership with the research team throughout the study. The OA QI (UK) supports the NICE quality standards for OA and they have been successfully used to assess the quality of OA consultations in primary care in the MOSAICS study. The OA QI (UK) has the potential for routine use in primary care to assess the quality of OA care provided to patients.

Ongoing research using both the UK and Norwegian OA-QI questionnaires is assessing the self-reported quality of OA care in different European populations.

**Electronic supplementary material:**

The online version of this article (doi:10.1186/s40900-016-0019-x) contains supplementary material, which is available to authorized users.

## Background

Osteoarthritis (OA) is a leading cause of joint pain and years lived with disability worldwide causing considerable detrimental impact on daily activities and quality of life [1–4]. OA is one of the main reasons for musculoskeletal consultations with a general practitioner by older adults [5].

High quality care is described as clinically effective, personal and safe, which is delivered to all users of a health service in all aspects of care [6]. However, previous studies have shown that the quality of care provided to patients with OA in primary care is suboptimal [7–11] and varies according to patient age and OA severity [12]. Research has shown that patients with OA need more information and education about the condition, diet, exercise, aids, and better support for self-management [13]. However, core recommended treatments such as exercise, weight loss and the provision of written information is underused for patients with OA [7]. Furthermore many core treatment are initiated by the patients themselves rather than doctor initiated [7, 14].

Several international guidelines exist which provide recommendations for the management of OA [3, 4, 15, 16]. International quality standards for OA have also been developed such as those recently published by the National Institute for Health & Care Excellence (NICE) [17], and the European Musculoskeletal Conditions Surveillance and Information Network (eumusc.net) [18]. Yet there are no robust or routinely collected measures used currently in general practice to monitor the quality of care for people with OA [19], although an OA e-template for use during consultations in primary care has recently been developed and tested [20, 21].

Quality indicators (QI) are ‘specific and measurable elements of practice that can be used to assess the quality of care’ [22]. They are used to assess care quality according to defined standards of care (e.g. NICE [17], eumusc.net [18]). QIs typically assess the processes of care given to patients [23] by measuring what the provider can offer patients and examining whether standards of care are being implemented.

A systematic review identified 15 QIs which are broadly applicable with current international guidance for the assessment of non-pharmacological and pharmacological management of OA in primary care [8]; however the authors recommended an increased use of QIs in primary care from the patient perspective.

The Management of OSteoArthritis In ConsultationS (MOSAICS) study [24] developed and evaluated a new model of supported self-management of OA to implement the NICE guidelines for OA in primary care [3]. The MOSAICS study aimed to evaluate the new model of supported self-management for OA in primary care, in terms of the quality of care from both a clinical and a patient perspective (see Additional file 1 for more information about the MOSAICS study). The findings of the MOSAICS study are subject to other papers in production. However, at the time of designing the MOSAICS study, there was a lack of evidence regarding the experiences of patients with OA in primary care and there were few appropriate quality indicators that captured the quality of primary care for OA from the patient perspective. Active and meaningful patient and public involvement (PPI) is increasingly viewed and encouraged as integral part of the research process to improve its quality and relevance [25–28]. Therefore the collaboration between the researchers and the RUG described in this article led to development of patient reported QIs for the MOSAICS model of self-support in primary care.

During the course of the MOSAICS study, the research team became aware of a questionnaire capturing the patient perspective of the quality of OA primary care being developed in Norway. The OsteoArthritis Quality Indicator (OA-QI) has since been validated for use to measure the quality of primary care for OA in a Norwegian population [29]. The Norwegian OA-QI, comprises 17 questions related to patient education and information, regular provider assessments, referrals, and pharmacologic treatment. The tool was developed by team of researchers and OA clinicians, with input from two patient partners who gave feedback on the content of the finalised questionnaire. The results of the validation study are reported elsewhere [29].

We report on the co-production of a set of self-report, RUG-generated QIs to capture the quality of primary care management of OA. We also compare the RUG-generated OA QI (UK) questionnaire with a Norwegian OA-QI questionnaire developed in parallel, leading to a final recommended OA QI (UK) questionnaire.

The manuscript was written using the Guidance for Reporting Involvement of Patients and Public (GRIPP) checklist for reporting Patient and Public Involvement (PPI) in research [30]. This checklist provides a structure for improving the quality of reporting of the PPI and is designed for studies that have included some form of patient and public involvement in research. The authors have referred to the checklist to ensure all the relevant aspects of PPI in this study were reported.

### PPI good practice

The PPI described in this article took place at the Arthritis Research UK Primary Care Centre, Keele University. This institution takes an explicit and systematic approach to involvement of patients and the public in research [31]. Formed in 2006, a Research User Group (RUG) was established to embed PPI across the whole of the Centre’s research activities and is supported by a dedicated PPI team and core funding. Currently, the RUG has over 60 members actively involved on over 60 projects, recruited on a basis of ‘expertise by experience’ of musculoskeletal and other long term conditions. Some RUG members have more experience in involvement in research than other members, though all provide the lay perspective of their health condition. Our approach to patient involvement draws on previous experience [32–35] and recommendations for the good practice of PPI [25, 30, 36, 37] so that RUG members can provide meaningful contributions to the research process (see Additional file 2 for more information about the RUG).

Our good practice principles include holding meetings in accessible venues at convenient times; allocated parking; meeting and greeting on arrival; training and support; inclusion of regular breaks during meetings; payment for RUG members’ time and contribution (if wanted); and reimbursement of expenses. Meetings between researchers and RUG members are made actively ‘jargon-free’ and any technical terms are explained in plain English.

## Methods

**Fig. 1:**
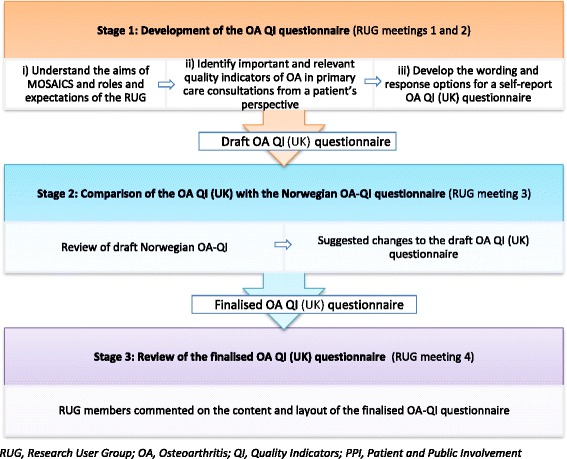
Overview of the stages of development of the OA QI

The MOSAICS study investigated whether a new way of supporting self-management, delivered during an OA consultation in primary care, could offer a clinically practical approach to implementing the core NICE recommendations [24]. We describe here the development of the OA QI (UK) questionnaire by the RUG group for use in the MOSAICS study in Stage 1, the comparison of this with a Norwegian OA-QI questionnaire in Stage 2, and review of the finalised OA QI (UK) questionnaire in Stage 3. Figure 1 provides an overview of the process.

### Patient and public involvement in this study

In 2009, RUG members with OA were invited to form an OA PPI group to work in partnership with researchers throughout the MOSAICS study, including the development of patient-reported QIs, set within a wider five year programme of research into OA [24, 38]. Furthermore, two members (AH: a former member of the wider Research User group and now PPI Support Worker/Coordinator; and RT: Lay member of the OA Research User Group) have co-authored this article, including writing the plain English and providing detailed comments on the manuscript prior to the final submission.

During the course of the MOSAICS study, members of the research team met with RUG members to co-produce the OA QI (UK) questionnaire for use in the MOSAICS study. The discussion meetings were facilitated by the Centre’s PPI Support Worker/Coordinator, the MOSAICS study Chief Investigator and a trial coordinator. The PPI Support Worker/Coordinator provided a key role by attend the meetings with RUG members to provide assistance and support, prior, during and after meetings. The MOSAICS study Chief Investigator (KD) has collaborated with the RUG on numerous research studies and is currently the senior academic lead for PPI in the Centre. All trial coordinators at the Centre have a responsibility for ensuring PPI in their respective studies and have lots of experience of collaborating with RUG members.

Discussion notes from the meetings were recorded on flip charts and in meeting minutes. Following each meeting, a summary of the outcomes and decisions written in plain English was sent to the RUG members to acknowledge their contribution and verify that all views had been captured. RUG members were also given the opportunity for further comment at the start of the next meeting.

It was not intended to formally evaluate the PPI interaction and the RUG members’ experience in the process. However, the impact of the RUG members is described in this article in the form of the co-produced OA QI (UK) questionnaire for use in the MOSAICS study.

Ethical approval for the PPI activities was not sought because the RUG members were acting as specialist advisers, providing valuable knowledge and expertise based on their experience of a health condition or public health concern and their involvement did not raise any ethical concerns [39]. However, the full MOSAICS research programme was approved by the North West 1 Research Ethics Committee, Cheshire, UK (REC reference: 10/H1017/76) [24].

### Stage 1: development of the OA QI questionnaire

Members of the OA RUG group (*n* = 10) were invited to a series of four discussion groups with the research team to develop the patient-reported QIs for patients with OA treated in primary care. The discussion groups took place over a three year period from 2009–2012. The objectives of the discussion groups were i) to understand the aims of MOSAICS and roles and expectations of the RUG members, ii) to identify important and relevant quality indicators for patients with OA when consulting in primary care, and iii) to develop wording and response options for a self-report OA QI (UK) questionnaire to assess the identified quality indicators (Fig. 1).

**Fig. 2:**
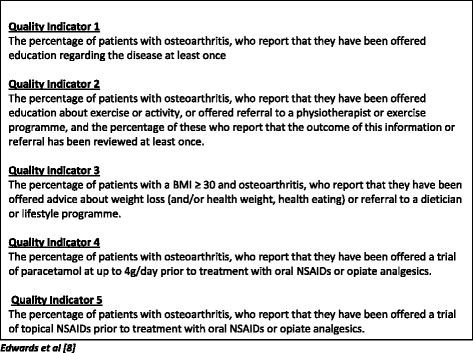
Five quality indicators identified from a systematic review used to stimulate discussion with the RUG members
iii) *Developing the wording and response options for a self-report OA QI (UK) questionnaire*
  Based on the list of important factors suggested by RUG members, an initial set of patient-reported OA QIs were generated. From this list, wording for questionnaire items to assess the QIs were drafted for an OA QI (UK) questionnaire. Over a further two meetings, RUG members and the research team worked together to refine and finalise the questions so they were suitable for use in a research trial. (See Fig. 3 for an outline of the process). Feedback and suggestions on the wording of the items and the scoring method (response options) were given on documents mailed to the RUG members between meetings.

i) *Understanding the aims of MOSAICS and roles and expectations of the RUG members*
  In the first meeting, a plain English summary of the MOSAICS study was introduced to set the context for the meetings and outline roles of the RUG members.
ii) *Identifying important and relevant quality indicators of OA in primary care consultations from a patient’s perspective*
  During facilitated discussions, RUG members identified factors they considered to be important to patients with OA consulting their general practitioner (GP) to help identify potential QIs for OA consultations. Each RUG member then ranked (1 to 5) the top five factors they considered the most important. Any factors not selected as ‘most important’ by at least one RUG member were excluded from further discussions.
  The research team then presented RUG members with five QIs identified from a previous systematic review [8] (Fig. 2). These QIs were selected on the basis of their relevance for the MOSAICS model OA consultation of supported self-management and were used to stimulate discussion within the RUG group. The QIs included whether during GP consultations, patients have been offered education and advice about their disease, exercise and weight loss, and offered pain relief in the form of paracetamol and topical (skin applied) non-steroidal anti-inflammatory drugs (NSAIDS). RUG members were asked to 1) consider each QI alongside the initial list of important factors when consulting their GP for OA and add other factors as necessary, and 2) suggest potential QIs and questions which could capture the quality of care from a patient perspective.

**Fig. 3:**
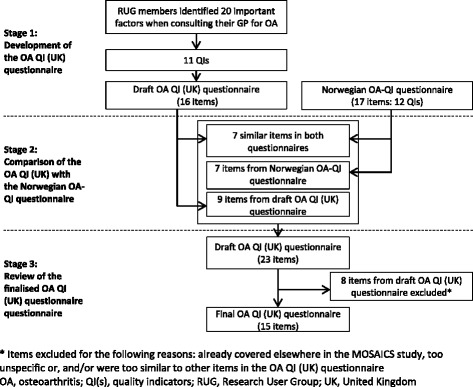
Overview of the OA QI (UK) questionnaire item development

### Stage 2: comparison of the OA QI (UK) with the Norwegian OA-QI questionnaire

The RUG group and MOSAICS study team had not planned to test the measurement (or psychometric) properties of the OA QI (UK) questionnaire. As mentioned earlier in this article, a similar OA QI questionnaire was being developed for use in primary care in Norway (the Norwegian OA-QI) [29].

In order to establish the measurement properties of the OA QI (UK) questionnaire, the Norwegian developers (NO, KH) produced an English translation of a draft version of the Norwegian OA-QI for item content and scoring comparison. RUG members reviewed this and compared its content with the OA QI (UK) questionnaire during the third meeting. Based on this comparison, RUG members suggested how the OA QI (UK) questionnaire could be refined and modified to include items in the validated Norwegian OA-QI. Where QIs in each questionnaire were similar, RUG members considered the appropriateness of the wording used in the Norwegian OA-QI for potential use.

### Stage 3: review of the finalised OA QI (UK) questionnaire

During the last meeting, the RUG reviewed, commented and suggested refinements to the OA QI (UK) questionnaire for primary care. RUG members were also asked to assess the face validity of the questionnaire by commenting further on its appearance and layout, ease of completion, and to identify anything ambiguous or difficult to understand. The research team also compared the content of the OA QI (UK) questionnaire with the other questionnaires and data collected in the MOSAICS to check for unnecessary duplication.

## Results

### Patient and public involvement

All of the RUG members (males 5, females 5) had OA and were aged 52–80 years. Four meetings were held during the three year period. All meetings were attended by between three and ten members.

### Stage 1: development of the OA quality indicators

#### Identifying important and relevant quality indicators of OA in primary care consultations from a patient’s perspective

RUG members initially discussed and identified 30 factors considered important and relevant to patients consulting their GP for OA. From this list, RUG members chose 20 factors as ‘most important’ (Table 1), which were grouped into the following domains: *information about OA; information about treatment for OA; information about self-management for OA; advice about using medications to relieve joint pain; information about exercise and activities; referrals to activity or exercise programmes, and the quality of the consultation with the GP*. These domains formed an initial list of seven patient-reported QIs for OA consultations. Following the review of the five quality indicators identified from a systematic review [8], an additional four QIs appropriate from a patient perspective (‘*patient has received a follow-up review of his/her joint problem’; ‘patient has received a referral for physiotherapy’; ‘patient has received advice about body weight and joint pain’, ‘patient has received a referral for weight loss services’*) were added. Therefore, 11 unique QIs were identified (Table 1).

**Table 1:** Selection of factors considered most important to patients with OA consulting their GP

Domain	Factors ranked most important to patients with OA consulting their GP (top 5 on a 1-5 scale)	Frequency	Draft Patient reported QIs for OA (‘In the last 3 months…..’)	Number of items in the draft OA QI (UK) questionnaire
Information about treatment for OA	Want to know about risks or side effects from the treatments	6	You have received information regarding treatment for joint problem from your surgery	1
	Want to know about pain management [medications]	4		
	Want to know about the expectations of any possible help, i.e. whether you need an X-ray/pain clinic/operation/investigations/scan/refer to a consultant	1		
	Want to know about surgery and also what to do when the consultant/surgeon says because of the cause there is nothing they can do	1		
	Want to get pain down to a level you can cope with	1		
Information about OA	What you want is an exchange between the GP and yourself	4	You have received information about your joint problem from your surgery	1
	How OA will affect quality of life	2		
	When you get more pain, you go to the GP	2		
	Pain has gone – will it stay that way?	1		
	Consultations are a combination of medical and patient expectations	1		
	Age means that OA is seen as inevitable	1		
	Want to know if it’s a serious condition	1		
Information about selfmanagement for OA	Want to know about techniques to [self] manage pain	2	You have received advice and support on how you might help yourself to manage or deal with your joint problem	3
Advice about using medications to relieve joint pain	How to best use anti-inflammatory medications	1	You have been offered advice about medications (to relieve joint pain)	4
Advice about exercise or activities	Want to know about should we be doing more or less	2	You have been offered information or advice on exercise or activity to help with your joint problem	3
Referral to exercise or activity programmes	Did certain things to help with quality of life (e.g. went to gym, go swimming)	3	You have been offered a referral to an exercise or activity programme for your joint problem	1
Quality of the consultation	The quality of consultation is important so that you know you shouldn’t just soldier on [live with pain]	3	You are satisfied with the overall quality of the consultation with his/her GP for OA^a^	0^a^
	Consultation to take a holistic approach/to ask patient about feelings and thoughts about the problem*	2		
	Previous consultation was a waste of time and I haven’t been back	1		
	Don’t want to waste the GP’s time	1		
Additional QIs following the discussion of the five treatment scenarios				
Follow up review	-	-	You have been given a follow-up review of your joint problem	2
Referral to physiotherapy	-	-	You have been offered a referral for physiotherapy for your joint problem	1
Advice about body weight	-	-	You have received advice about body weight and joint pain	0^b^
Referral to weight loss services	-	-	You have received a referral for weight loss services	0^b^

^a^Questionnaire items not created for this QI as it was adequately captured elsewhere in the larger MOSAICS survey

^b^Questionnaire items not created for these two QIs as they were initially captured elsewhere in the larger MOSAICS study

#### Developing the wording and response options for a self-report OA QI (UK) questionnaire

Using the list of eleven self-reported QIs for OA consultations, RUG members suggested questionnaire wording and response options to assess each QI. RUG members stated that the 3-month recall period, as determined by the MOSAICS trial design, was an appropriate period to have had at least one consultation with their GP for a joint problem. They also suggested that the response options should be a simple 3-level response format for all questions for the draft questionnaire: “Yes”, “No”, and “Don’t know”.

The RUG members’ suggestions were drafted into a questionnaire. Views of the overall quality of the consultation were captured in other parts of the MOSAICS study, so this item was not included in the questionnaire. To avoid multiple dimensions of care within a single question, one QI (advice about using medications to relieve joint pain) was split into four questions, two QIs (information about self-management for OA; advice about exercise or activities) into three items, and one other QI (follow up review) was split into two questions, respectively.

The resultant draft OA QI (UK) questionnaire comprised 16 items. In the second group meeting, RUG members reviewed the draft questionnaire and worked with the research team to refine its content. RUG members provided further comments on the ease of understanding and relevance of the questions. They suggested wording for the questionnaire instructions and changes to improve the clarity, specificity and order of the questions. RUG members suggested changing the wording for the ‘don’t know’ response option where relevant if the respondent could not remember, if they had not received an aspect of care, or if the question was not applicable.

### Stage 2: comparison of the OA QI (UK) with the Norwegian OA-QI questionnaire

After reviewing both questionnaires, RUG members suggested that the draft OA QI (UK) questionnaire and the draft Norwegian OA-QI were similar. Seven items in the draft OA QI (UK) questionnaire capturing six quality indicators: (*information about OA, information about treatment for OA, information about self-managing OA, advice about exercise or activities for OA, referral to exercise or activity programmes for OA, advice about the use of medications to relieve joint pain*) used comparable wording to those included in the draft Norwegian OA-QI (Table 2). Of these, RUG members and the research team agreed to retain the wording used in the draft OA QI (UK) questionnaire for three items and adopt all or some of wording in the draft Norwegian OA-QI for the other four items for the final questionnaire. Both questionnaires used similar three-level response options.

**Table 2:** Comparison of the draft OA QI (UK) and Norwegian OA-QI questionnaires

Quality Indicator	Draft OA QI (UK)	Draft Norwegian OA-QI (first English translation)	Published Norwegian OA-QI (tested for validity and reliability)^a^	RUG and research team’s recommendations for final OA QI (UK) (✓ = retain or add; X = not required)	RUG and research team’s recommendations (Changes to wording for final OA QI (UK))
Patient has received information about OA	*Have you been given any information about your joint problem(s) from your surgery?*	*Have you been informed about how the disease naturally evolves?*	*Have you been given information about how the disease usually develops over time?*	✓	Retain (with draft OA QI (UK) wording and ‘written or verbal’ information included)
Patient has received information about treatment for OA	*Have you been given any information regarding treatment for your joint problem(s) from your surgery?*	*Have you been informed about treatment?*	*Have you been given information about different treatment alternatives?*	✓	Retain (Adopt draft Norwegian OA-QI wording)
Patient has received information about self-managing OA (advice)	*Have you been given any advice on how you might help yourself to manage or deal with your joint problem(s)?*	*Have you been informed about self-management?*	*Have you been given information about how you can live with the disease?*	✓	Retain (with draft OA QI (UK) wording)
Patient has received information about self-managing OA (support)	*Have you been given any support on how you might help yourself to manage or deal with your joint problem(s)?*	-	-	✓	Retain (with draft OA QI (UK) wording)
Patient has received information about self-managing OA (support)	*Have you been given any support from your ‘surgery’ or other health care professionals e.g. physiotherapists or occupational therapists to help you manage your joint problem(s)?*	-	-	X	Not required
Patient has received a follow up review	*Have you been given a follow up review of your joint problem(s) at least once?*	-	-	X	Not required
Patient has received a follow up review	*Have you been given a follow up review of your joint problem(s) ever?*	-	-	X	Not required
Patient has received advice about changing lifestyle	-	*Have you been offered advice about lifestyle change?*	*Have you been given information about how you can change your lifestyle?*	X	Not required
Patient has received advice about exercise or activities for OA (current participation in exercise)	*Do you participate in any exercise?*	-	-	X	Not required
Patient has received advice about exercise or activities for OA	*Have you been offered information or advice on exercise or physical activity to help you with your joint problem(s)?*	*Have you been informed about the impact of muscle strengthening or aerobic exercise programs?*	*Have you been given information about the importance of physical activity and exercise?*	✓	Retain (with draft OA QI (UK) wording but ‘muscle strengthening’ included)
Patient has received a referral to exercise or activity programmes for OA	*Have you been offered a referral for exercise or activity programme for your joint problem(s) (e.g. tai chi, swimming, keep fit)?*	*Have you been referred to services for a directed or supervised strengthening or aerobic exercise programs?*	*Have you been referred to someone who can advise you about physical activity and exercise (e.g. a physiotherapist)?*	✓	Adopt draft Norwegian OA-QI wording
Patient has received referral about exercise or activities for OA (exercise programme suggested)	*Has an exercise or activity programme been suggested to help you manage with your joint problem(s)?*	-	-	X	Not required
Patient has received advice about body weight and joint pain	-	*Have you been advised to lose weight, if you are overweight and obese?*	*If you are overweight, have you been advised to lose weight?*	✓	Adopt draft Norwegian OA-QI wording (‘if you are overweight’ removed)
Patient has received a referral for weight loss	-	*Have you been referred to services for losing weight, if you are overweight and obese?*	*If you are overweight, have you been referred to someone who can help you to lose weight?*	✓	Adopt draft Norwegian OA-QI wording (‘if you are overweight’ removed and examples of weight loss services added)
Patient has received a referral for an assessment of activities of daily living		*If you have had problems related to activities of daily living, have these problems been assessed in the last year?*	*If you have had problems related to daily activities, have these problems been assessed by health personnel in the last year?*	X	Not required
Patient has received a referral for physiotherapy	*Have you been offered a referral for physiotherapy for your joint problem(s)?*			X	Not required
Patient has received a referral for an assessment for aids for daily living	-	*If you have problems related to other activities of daily living*, *has your need for assistive devices (e.g. splints, assistive technology for cooking or personal hygiene) been assessed?*	*If you have problems related to other daily activities*, *has your need different appliances and aids been assessed (e.g. splints, assistive technology for cooking or personal hygiene, a special chair)?*	✓	Adopt draft Norwegian OA-QI wording (‘assistive devices’ changed to ‘appliances and aids to daily living’)
Patient has received a referral for an assessment for walking aids	-	*If you have problems related to walking, has your need for ambulatory assistive devices (e.g. stick, crutch, or walker) been assessed?*	*If you have problems with walking, has your need for a walking aid been assessed (e.g. stick, crutch, or walker)?*	✓	Adopt draft Norwegian OA-QI wording (‘ambulatory assistive devices’ changed to ‘a walking aid’)
Patient has received as assessment of his/her pain	-	*If you have pain, has your pain been assessed in the last year?*	*If you have pain, has it been assessed in the past year?*	X	Not required
Patient has received advice about the use of medications to relieve joint pain	*Have you been offered advice by your surgery about taking paracetamol before taking other tablets?*	*If you have pain, was paracetamol the recommended pharmacological therapy for your osteoarthritic pain?*	*If you have pain,* was *acetaminophen the first medicine that was recommend for your osteoarthritic pain?*	✓	Adopt draft Norwegian OA-QI wording (‘pharmacological’ and ‘osteoarthritic’ removed)
Patient has received advice about the use of medications to relieve joint pain	*Have you been offered advice by your surgery about taking the following medication: paracetamol?*	-	-	X	Not required
Patient has received advice about the use of medications to relieve joint pain (Recommendation for stronger analgesia)	-	*If you have prolonged severe pain, for which paracetamol does not provide pain relief, have you been offered stronger analgesic drugs (e.g.,…)?*	*If you have prolonged severe pain, which is not relieved sufficiently by paracetamol, have you been offered stronger pain killers (e.g., coproxamol, co-dydramol, tramadol, co-codamol, dihydrocodeine, codeine)?*	✓	Adopt draft Norwegian OA-QI wording (‘analgesic’ changed to ‘painkilling’)
Patient has received advice about the use of medications to relieve joint pain (Anti-inflammatory drug information)	*Have you been offered advice by your surgery about taking the following medication: topical anti-inflammatory creams or gels (e.g. Votarol gel, diclofenac, ibuprofen cream*)?	*If you use anti-inflammatory drugs (e.g. …), have you received information about the effects and potential side effects associated with this drug?*	*If you are taking antiinflammatory drugs, have you been given information about the effects and possible side effects of this medicine (e.g., ibuprofen, Nurofen, Brufen, diclofenac, Voltarol, naproxen, Naprosyn, Celebrex)?*	✓	Adopt draft Norwegian OA-QI wording
Patient has received advice about the use of medications to relieve joint pain (capsaicin cream)	*Have you been offered advice by your surgery about taking the following medication: capsaicin cream*?	-	-	X	Not required
Patient has received advice about the use of medications to relieve joint pain (Consideration for corticosteroid injection)	-	*If you have experienced an acute deterioration in symptoms, has a corticosteroid injection been considered?*	*If you have experienced an acute deterioration of your symptoms, has a corticosteroid injection been considered?*	✓	Adopt draft Norwegian OA-QI wording
Patient has been considered for referral for surgery	-	*If you experience severe symptomatic osteoarthritis, and pharmacological therapy and exercises have no response, have you been referred for evaluation of surgery (e.g., total joint replacement)?*	*If you are severely troubled by your osteoarthritis, and exercise and medicine do not help, have you been referred and assessed for an operation (e.g., joint replacement)?*	✓	Adopt draft Norwegian OA-QI wording

^a^Final wording of Norwegian OA-QI was published after the OA RUG group reviewed and compared the draft OA QI (UK) with the draft Norwegian OA-QI

RUG members recommended that a further seven items (capturing six QIs) included in the Norwegian OA-QI were relevant and should be added to the UK questionnaire. They suggested minor changes to wording to make them more appropriate for the UK (Table 2). Three other items from the Norwegian OA-QI questionnaire (*advice about changing lifestyle; assessment of daily activities; assessment of pain*) were captured elsewhere in the MOSAICS study and therefore not required for the OA QI (UK) questionnaire. Nine items from the draft OA QI (UK) that were not present in the Norwegian OA-QI were retained at this stage. Therefore, at the end of Stage 2, the draft version of the OA QI (UK) questionnaire contained 23 items (Fig. 3).

### Stage 3: review of the finalised OA QI (UK) questionnaire

The iterative process of redrafting and reviewing the questionnaire continued into the fourth meeting until the RUG members and researchers agreed on the final draft version. Along with subtle changes to item wording suggested by RUG members, the research team and RUG members agreed to retain one item from the draft OA QI (UK) (on *support for self-managing OA*). Eight items from the draft OA QI (UK) (*support from ‘surgery’ or other health care professionals to help you manage your joint problem; follow up review received (2 items); current participation in exercise; exercise programme suggested; referral for physiotherapy; advice about taking paracetamol received; advice about taking capsaicin cream received*) were either covered elsewhere in the MOSAICS study [24], too generic or were too similar to other items (Table 2). Therefore, these eight items were not included in the final 15-item OA QI (UK) questionnaire (see Additional file 3). RUG members and researchers agreed that the length of the final version of the questionnaire was appropriate to capture important quality indicators of OA in primary care consultations from a patient’s perspective without overburdening those who complete it.

## Discussion

This study describes the development of patient-reported quality indicators questionnaire for the primary care of osteoarthritis, which were derived by members of a Research User Group, working in partnership with researchers. The OA QI (UK) has been successfully used in a large randomized control trial of a new model of supported self-management of OA (the MOSAICS study) [22] and a study to audit the quality of OA primary care practice in the United Kingdom, Norway, Denmark and Portugal. While the full results of these studies are subject to other papers in production, the focus of this article is on the role and impact of PPI to develop the OA QI (UK).

The active, meaningful and on-going involvement of patients as partners in the research process is a strength of this study. The perspectives of patients may differ from the perspectives of healthcare professionals or information recorded by professionals in medical records [40, 41]. Therefore, the unique perspectives of patients with OA based on their experience of the condition and past consultations in primary care has enhanced the development of patient-centred quality indicators for use in OA primary care. We acknowledged that the PPI input in this study incorporated the perspectives of a small group of patients, as small as three people for one meeting. Also, the RUG membership was not greatly diverse, in terms of age, ethnicity, and physical abilities. While obtaining a range of perspectives is the objective of PPI in research and not necessarily ‘representativeness’, it is possible however that the OA QI (UK) does not cover the full range of quality indicators relevant to the population of patients with OA. Nevertheless, the sequential and iterative development of the OA QI (UK) allowed the researchers and RUG members to review and critique earlier suggestions made by the RUG.

The RUG group identified important factors related to the quality of OA care provided by a primary care healthcare professional and suggested item wording for a questionnaire. By comparing the OA QI (UK) questionnaire with a similar one developed in Norway, the RUG members helped redraft and refine the final questionnaire. The RUG collaborated with the research team throughout the development of the OA QI (UK) but were also involved on other aspects of the MOSAICS study such as developing a self-management guidebook for patients with OA and participant information sheets [33, 42]). Regular meetings were set up for these. Though there were extended gaps between meetings regarding the OA QI (UK) development, the timings of the meetings were governed by the MOSAICS study timeline. However, RUG members were provided with feedback of the meeting and given the opportunity to comment. This process built upon existing working relationships and trust between the RUG and researchers.

The research team embraced the contribution of the RUG members and implemented many of their suggestions. The concepts included in the finalised OA QI (UK) were generated by the RUG members. Working in collaboration the RUG members and the research team shaped the items in survey questions suitable for use in a research trial. The Chief Investigator did not make decisions on the final content of the questionnaire without the fully informed RUG and explained if information was already captured elsewhere in the MOSAICS study. For example, RUG members did identify eight other important and relevant QIs not included in the final version of the OA QI (UK). So, to avoid repetition and participant burden in the MOSAICS study, these eight items were not included in the final 15-item OA QI (UK) questionnaire. These decisions were fully explained to the RUG members. Therefore, the RUG members' contribution ensured that the resulting OA QI (UK) incorporated issues relevant to patients with OA, written in a language that patients found easy to understand.

The OA QI (UK) was developed to assess the uptake of treatment recommended by NICE [3] and complements the new NICE Quality Standards of Care for OA [15]. Using evidence from a systematic review [8], the OA QI (UK) is a 15-item questionnaire that covers treatments offered by healthcare professionals in primary care. The OA QI (UK) supports the recently published NICE Quality Standards of Care for OA [17] and the European Musculoskeletal Conditions Surveillance and Information Network (eumusc.net) recommendations for the OA Standards of Care across European member states [43]. For example, it captures five of the eight NICE quality statements either fully or partially from a patient’s viewpoint. The OA QI (UK) and the Norwegian OA-QI also sit alongside established outcome measures of OA management and provide process measures of the quality of OA care. The eumusc.net has developed OA health care quality indicators (HCQI-OA) and an accompanying audit tool for clinicians and health care providers [44]. The OA QI (UK) and HCQI-OA both include six similar quality indicators. This questionnaire, if used in routine practice, will have the patients’ perspectives embedded in an evaluation of care quality. Though developed for use across primary care settings, the OA QI (UK) may be further refined to meet the specific needs and priorities of local health care settings, if required.

Establishing the measurement properties of a questionnaire is an important step in its development. The use of scientifically sound and decision-relevant measures allows the collection of evidence on the benefits of intervention (or care practices) from a patients’ perspective [45]. The OA QI (UK) and the Norwegian OA-QI were developed in parallel. Given the similarity between the construct and wording of the two questionnaires and the direct additions of items from the Norwegian OA-QI, the UK version ‘adopted’ the measurement properties of the validated Norwegian version. The comparison of the questionnaires used a translated, draft version of the Norwegian OA-QI. However, the Norwegian OA-QI was further refined with some changes to the item wording before validity and reliability testing. Though they are very similar in content and wording, the finalised, validated Norwegian OA-QI was published after the OA QI (UK) was produced and implemented in the MOSAICS study. The measurement properties of the OA QI (UK) was not tested because the 14 (out of the 15 items) were identical or contained subtle changes in wording to items in the validated Norwegian questionnaire. Therefore, conducting a full validation study on the OA QI (UK) questionnaire was not justified at this stage. However, the assumption that the measurement properties of the two questionnaires are similar may need further exploration.

The overall positive feedback from RUG members on the Norwegian OA-QI enabled adaptations to the OA QI (UK) to be made with confidence. The development of the both questionnaires was coincidental. Although there was differences in how they were developed (one mainly patient-led and the other mainly researcher/clinician derived), this study has demonstrated that patients and researchers have similar expectations about what constitutes good quality care in OA in different European countries. It also highlights the value of the active, meaningful and useful contribution of patients in the research process. Furthermore, the consistency of quality indicators for OA consultations in two European countries has now provided a unique opportunity to compare QIs across European countries [46]. This may lead to the development of a single, combined questionnaire for use in routine clinical practice to assess the quality of OA care provided to patients.

## Conclusion

This study has demonstrated that active involvement of patients in research, working in partnership with researchers, identified important and relevant OA quality indicators, and developed a self-reported questionnaire to measure them. The OA QI (UK) questionnaire aligns with current national and international standards and process measures of OA care (e.g. NICE, eumusc.net) and is consistent with quality indicators validated for Norwegian OA consultations. The development of two OA quality indicator questionnaires was coincidental but has led to further research to compare patient-reported OA QIs across European countries. Following this work, a single refined OA QI questionnaire for use in routine clinical practice is planned.

## Additional files

Additional file 1: **Overview of the Managing Osteoarthritis in Consultations (MOSAICS) study.** (DOCX 12 kb)

Additional file 2: **Overview of Patient and Public Involvement in the Institute of Primary Care and Health, Keele University.** (DOCX 12 kb)

Additional file 3: **The Osteoarthritis Quality Indicators (UK) Questionnaire.** (DOCX 56 kb)

## Abbreviations

eumusc.net: European musculoskeletal conditions surveillance and information network
GP: general practitioner
GRIPP: guidance for reporting involvement of patients and public
HCQI-OA: health care quality indicators for osteoarthritis
MOSAICS: Management of OSteoArthritis In ConsultationS
NICE: National Institute for Health and Care Excellence
OA: osteoarthritis
PPI: patient and public involvement
QI(s): quality indicator(s)
RUG: research user group
UK: United Kingdom
