# Supplementary material for: Patient-reported quality indicators for osteoarthritis: a patient and public generated self-report measure for primary care
Source: Res Involv Engagem. 2016 Mar 17;2:5. doi: 10.1186/s40900-016-0019-x (PMC5611660; doi:10.1186/s40900-016-0019-x)
Supplement: Supplementary file 1 — Overview of the Managing Osteoarthritis in Consultations (MOSAICS) study. (DOCX 12 kb) [file 40900_2016_19_MOESM1_ESM.docx]

**Additional File 1. The Managing Osteoarthritis in Consultations (MOSAICS) study**

The work described in this paper was a component of the Managing Osteoarthritis in Consultations (MOSAICS) trial, an investigation of the feasibility, acceptability and impact of implementing the National Institute for Health and Care Excellence (NICE) osteoarthritis (OA) Guideline. There is as yet no evidence on the feasibility of implementing recommendations from the NICE OA guidelines in primary care, or of the effect these recommendations have on the condition.

The main aim of the MOSAICS study was to test a complex patient-focused intervention: information for patients, trained health care professionals responsive to patients’ needs, and model consultations for OA. The study will determine the clinical and cost effectiveness of a model OA consultation (MOAC), implementing the core recommendations from the NICE OA guidelines in primary care. Secondary aims included describing the uptake of core NICE OA recommendations using quality indicators of care in participants aged 45 years and over with joint pain.

The innovations in the trial intervention were: i) an OA Guidebook developed with patients and health professionals to provide patient-centred and evidence-based information, ii) a model OA consultation with GPs and practice nurses, and iii) access to a practice-based nurse-led OA clinic (providing support for self-management).

The design is a mixed methods study with a nested cluster randomised controlled trial.

The main output from the study will be to determine whether the MOAC intervention is clinically and cost effective. Additional outputs will include resources for patients and professionals regarding supported self-management and uptake of NICE guidance.
