# Supplementary material for: Patient-reported quality indicators for osteoarthritis: a patient and public generated self-report measure for primary care
Source: Res Involv Engagem. 2016 Mar 17;2:5. doi: 10.1186/s40900-016-0019-x (PMC5611660; doi:10.1186/s40900-016-0019-x)
Supplement: Supplementary file 2 — Overview of Patient and Public Involvement in the Institute of Primary Care and Health, Keele University. (DOCX 12 kb) [file 40900_2016_19_MOESM2_ESM.docx]

**Additional File 2. Patient and Public Involvement in the Institute of Primary Care and Health, Keele University**

The Research User Group (RUG)

Involving and engaging the public is at the heart of the research conducted by the Institute of Primary Care and Health Sciences at Keele University. Patient and Public Involvement (PPI) has changed the way we do research. Since the formation of our Research User Group (RUG) in 2006, we have actively involved patients and the public across all of our research activities.

RUG members are recruited on the basis of ‘expertise by experience’ of musculoskeletal and other long-term conditions. Now with over 60 members involved in as many studies, the RUG is supported by two dedicated part-time staff, both with experience of musculoskeletal and long-term conditions; a frontline adviser from the Research Design Service; and an Academic Lead from the Institute’s Professoriate. Our PPI team has developed a number of guides to further support the involvement of the RUG members. Along with research grant funding to support PPI within studies, our Arthritis Research UK Centre of Excellence Award and the Primary Care Consortium Board provide core funding for our lay members.

The impact of PPI is evident throughout the research cycle, from initiating and shaping new ideas, to research, designing and managing studies, through to helping to disseminate and implement the Institute’s research. Key to the meaningful and long term involvement of lay people in the Institute’s research is its leadership and organisational commitment to patient and public involvement. (Jinks et al. J Care Serv Manag. 2013;7(4):14654).
