# Supplementary material for: Patient-reported quality indicators for osteoarthritis: a patient and public generated self-report measure for primary care
Source: Res Involv Engagem. 2016 Mar 17;2:5. doi: 10.1186/s40900-016-0019-x (PMC5611660; doi:10.1186/s40900-016-0019-x)
Supplement: Supplementary file 3 — The Osteoarthritis Quality Indicators (UK) Questionnaire. (DOCX 56 kb) [file 40900_2016_19_MOESM3_ESM.docx]

**Additional File 3 The Osteoarthritis Quality Indicators (UK) Questionnaire**

**Section A. Your recent GP consultation**

The following questions ask about any treatment or care you may have received for your joint problem(s) or joint pain(s). If you haven’t received any we would still be interested in your answers.

There are different treatment alternatives for people with joint problems. We would like to know what treatment, information or advice you have received **from your GP surgery** **in the last 3 months** in relation to your joint problems. Please put only one cross for each question.

| (***Please cross one box only on each line)*** | **Yes** | **No** | **Don’t remember** |
| --- | --- | --- | --- |
| 1. Have you been given any written or verbal **information** about your joint problem(s)? |  |  |  |
| 1. Have you been informed about treatment? |  |  |  |
| 1. Have you been given any **advice** on how you might help yourself to manage or deal with your joint problem(s)? |  |  |  |
| 1. Have you been given any **support** on how you might help yourself to manage or deal with your joint problem(s)? |  |  |  |
| 1. Have you been offered **information or advice** about exercise, muscle strengthening or physical activity to help you with your joint problem(s)? |  |  |  |
| 1. Have you been offered **a referral** to services for a directed or supervised strengthening or physical activity programme? |  |  |  |
|  | **Yes** | **No** | **Not overweight** |
| 1. Have you been advised to lose weight? |  |  |  |
| 1. Have you been offered **a referral** to services for losing weight, for example a dietician, or weight watchers? |  |  |  |
|  | **Yes** | **No** | **No such problems** |
| 1. If you have problems related to walking, has **your need** for a walking aid (e.g. stick, crutch or walker) **been assessed**? |  |  |  |
| 1. If you have problems related to other activities of daily living, has **your need** for appliances and aids to daily living (e.g. splints, assistive technology for cooking or personal hygiene) **been assessed**? |  |  |  |

**(continued)**

| The following questions ask about any treatment or care you may have received for your joint problem(s) or joint pain(s) **in the last 3 months.** | | | |
| --- | --- | --- | --- |
| (***Please cross in one box only on each line)*** | **Yes** | **No** | **No pain** |
| 1. If you have pain, was paracetamol the recommended therapy for your pain? |  |  |  |
| 1. If you have prolonged severe pain, for which paracetamol does not provide pain relief, have you been offered stronger painkilling drugs? (e.g. Co-dydramol, Tramadol, Co-codamol, Dihydrocodeine, Codeine, Co-proxamol) |  |  |  |
| 1. If you use anti-inflammatory drugs (e.g. Ibuprofen, Nurofen, Brufen, Diclofenac, Voltarol, Naproxen, Naprosyn, Celebrex), have you received information about effects and potential side effects associated with this drug? |  |  |  |
| 1. If you have experienced an acute deterioration in symptoms, has a corticosteroid joint injection been considered? |  |  |  |
|  | **Yes** | **No** | **Not Severe**  **symptoms** |
| 1. If you experience severe symptomatic osteoarthritis and pharmacological therapy and exercises have no response, have you been referred for evaluation of surgery (e.g. total joint replacement)? |  |  |  |
